# Physicochemical and Structural Characteristics of Date Seed and Starch Composite Powder as Prepared by Heating at Different Temperatures

**DOI:** 10.3390/polym17141993

**Published:** 2025-07-21

**Authors:** Muna Al-Mawali, Maha Al-Khalili, Mohammed Al-Khusaibi, Myo Tay Zar Myint, Htet Htet Kyaw, Mohammad Shafiur Rahman, Abdullahi Idris Muhammad, Nasser Al-Habsi

**Affiliations:** 1 Department of Food Science and Nutrition, College of Agricultural and Marine Sciences, Sultan Qaboos University, P.O. Box 34-123, Al-Khodh 123, Muscat, Oman; s118049@student.squ.edu.om (M.A.-M.); mahaalikh@gmail.com (M.A.-K.); mohamedk@squ.edu.om (M.A.-K.); shafiur@squ.edu.om (M.S.R.); abdullahimuhammad300@gmail.com (A.I.M.); 2 Department of Physics, College of Science, Sultan Qaboos University, P.O. Box 36-123, Al-Khod 123, Muscat, Oman; m.myint@squ.edu.om; 3 Nanotechnology Research Center, Sultan Qaboos University, P.O. Box 33-123, Al Khod 123, Muscat, Oman; htet@squ.edu.om

**Keywords:** date seed powder, starch composite, solubility, crystallinity, functional fiber

## Abstract

Date seeds, a by-product of the pitted-date industry, are often discarded as waste. This study investigated the interaction between date seed powder and starch at different concentrations (0, 1, 5, 10, and 20 g/25 g composite) and temperatures (40 °C and 70 °C). The results revealed that the hygroscopicity of date seed powder (9.94 g/100 g) was lower than starch (13.39 g/100 g), and its water absorption (75.8%) was also lower than starch (88.3%), leading to a reduced absorbance capacity in composites. However, the solubility increased with a higher date seed content due to its greater solubility (17.8 g/L) compared to starch (1.6 g/L). A morphological analysis showed rough, agglomerated particles in date seed powder, while starch had smooth, spherical shapes. This study also found that the composites formed larger particles at 40 °C and porous structures at 70 °C. Crystallinity decreased from 41.6% to 12.8% (40 °C) and from 24.0% to 11.3% (70 °C). A thermal analysis revealed three endothermic peaks (glass transitions and solid melting), with an additional oil-melting peak in high-seed samples. FTIR spectra showed changes in peak intensities and locations upon seed incorporation. Overall, these findings revealed that, the incorporation of date seed powder–starch composites into bakery formulations offers a promising strategy for developing fiber-enriched products, positioning them as functional ingredients with added nutritional value.

## 1. Introduction

Date palm (*Phoenix dactylifera* L.) is one of the principal fruit crops cultivated globally, resulting in a substantial surplus production in various regions [1]. The global production of date fruits has shown a steady upward trend, reaching approximately 9.82 million metric tons in 2023, up from 8.4 million metric tons in 2017 [2]. Date seeds, also referred to as date pits, constitute 10–15% of the total fruit weight, depending on the cultivar and fruit quality [3]. These seeds are by-products of the pitted-date industry and are commonly discarded or, in some instances, used as feed for livestock such as cattle, sheep, camels, and poultry [4,5,6]. In the Arab region, they have been traditionally employed to produce a caffeine-free beverage [7], and recent research has explored their potential use in food systems, such as substituting vegetable oils in mayonnaise [8], as well as in cosmetic formulations, including body creams, shampoos, and soaps [3]. However, these applications remain at the exploratory stage and have not achieved commercial viability.

The proximate composition of date seeds varies slightly across cultivars; however, they are generally recognized for their high dietary fiber content, typically exceeding 70% [9]. Major fiber components include cellulose (20.0–46.8 g/100 g), hemicellulose (17.5–55.0 g/100 g), lignin (11.0–30.6 g/100 g) [6,10,11], and soluble fiber (4.4–6.7 g/100 g) [12], alongside notable amounts of protein (up to 12.5 g/100 g) and lipids (3.9–13.8 g/100 g) [13,14,15,16,17]. Furthermore, date seeds are recognized for their potent antioxidant activity, attributed to their high phenolic content (3942 mg/100 g) and total antioxidant capacity (80,400 μmol/100 g) [18]. These nutritional and bioactive attributes render date seeds a promising candidate for incorporation into starch-based matrices aimed at enhancing the structural, thermal, and functional characteristics of food and packaging materials.

Several studies have examined the incorporation of date seed fibers into bakery products such as bread, biscuits, and cakes, as well as dietary supplements [19,20]. For instance, Ghasemi et al. [20] demonstrated that incorporating 5% or 10% date seed powder into a sponge cake significantly affected (*p* < 0.05) its textural attributes, antioxidant capacity, fiber and protein content, fat content, mineral composition, and sensory properties. Similarly, Halaby et al. [21] reported that pan bread fortified with 15% date seed powder received the highest sensory acceptance scores compared to control and other formulations. Beyond food applications, date seed fibers have been investigated as bio-fillers in edible and biodegradable composites designed for food preservation, oil uptake reduction during deep frying, and shelf-life extension [9]. In these applications, the fibers are typically blended with natural polymers, such as starch, cellulose, and alginate, often in conjunction with crosslinkers and plasticizers [22,23,24]. Although numerous studies have confirmed the potential of natural fibers to enhance the tensile strength, moisture resistance, and thermal behavior of starch-based films [25,26,27], relatively few have focused specifically on date seed fibers [28]. Moreover, data on starch-based composites formulated exclusively with unmodified date seed powder, without the addition of synthetic crosslinking or plasticizing agents, remain scarce.

The present study addresses this critical knowledge gap by evaluating the physicochemical, structural, and thermal properties of starch–date seed composites formulated without any synthetic additives. The novelty of the current research lies in its investigation of date seed powder as a standalone, natural additive for starch matrices, contributing to the advancement of clean-label, sustainable food systems and biodegradable materials. The abundant availability of date seeds [29], their high content of insoluble dietary fiber [30], and their antioxidant richness [31] collectively support their candidacy for functional food packaging and edible applications [32,33,34].

Despite their nutritional potential, date seeds remain underutilized in the food industry. Their high insoluble fiber content can limit their direct applicability; however, this limitation may be overcome by combining them with other fiber sources to improve their physicochemical behavior and facilitate their inclusion in a wider range of food products. At present, there remains a substantial gap in understanding how unmodified date seed powder interacts with key food biopolymers, particularly under thermal conditions relevant to food processing.

Temperature is a crucial factor affecting the structural and functional integrity of biopolymer composites during processing, storage, and application. It can influence crystallinity, morphology, water interaction, and thermal resistance. The temperatures selected for this study (40 °C and 70 °C) represent common processing and handling conditions. The former simulates a moderate thermal exposure, such as low-temperature drying, while the latter reflects more intense conditions, like pasteurization, baking, or heated coating applications [32,35,36]. Investigating the composite behavior at both temperatures enables a comprehensive understanding of their thermal adaptability and performance under realistic scenarios.

Accordingly, the primary objective of this study is to investigate the physicochemical and structural interactions between starch and date seed powder under thermal treatments at 40 °C and 70 °C, without the use of synthetic additives. This study evaluates the following: (i) proximate composition; (ii) solubility, water absorption, and hygroscopicity; (iii) surface morphology using scanning electron microscopy (SEM); (iv) crystallinity through X-ray diffraction (XRD); (v) thermal transitions via differential scanning calorimetry (DSC); and (vi) functional group mobility through Fourier-transform infrared spectroscopy (FTIR).

By providing a comprehensive analysis of the functional and structural performance of starch–date seed composites, this study offers valuable insights into the development of sustainable and biodegradable food components. The findings contribute foundational knowledge for utilizing agricultural by-products, such as date seeds, in value-added applications, including baked goods, ready-to-eat snacks, meat analogs, dairy alternatives, and edible packaging. This work thus supports both waste valorization and innovation in the formulation of clean-label functional food systems.

## 2. Materials and Methods

### 2.1. Materials

Date seeds (*Mebsily* variety) were purchased from a local farmer in Bidiya, Oman. They were soaked in water for 2 h, washed under running water to remove any excess adhering date flesh, and then dried in a hot-air drier (Sanyo Gallenkamp, Cambridge, UK) at 60 °C for 72 h. The dried date seeds were then ground into a fine powder using a heavy-duty grinder (Foss, Hoganas, Sweden). The fine date seed powder was sieved through a mesh of 125 μm size and stored in amber-sealed bottles at 25 °C. The date seed powder was placed in a jar with saturated salt solution (i.e., lithium chloride), maintaining the relative humidity at 11.3%. The jar contained thymol to prevent mold growth in the sample. The jar was left at room temperature (25 °C) until it reached equilibrium. Analytical-grade potato starch (Sigma-Aldrich, St. Louis, MO, USA) was also equilibrated as mentioned above and used in the composite formulation; according to the established literature, native potato starch generally exhibits an amylose-to-amylopectin ratio of about 25:75 [36,37]. The higher amylopectin content, characterized by its highly branched structure with (1–6) glycosidic bonds, enhances the film-forming ability, flexibility, and thermal stability of starch-based composites. These characteristics make potato starch particularly suitable for developing biodegradable films and composites for food packaging and fortification applications [38]. Additionally, its functional properties, such as thickening, stabilizing, and moisture retention, underscore its potential as an effective food additive in composite formulations [39,40,41].

### 2.2. Combination of Date Seed with Starch

Date seed powder (i.e., 0, 1, 5, 10, 20 g) was mixed with analytical grade starch (Sigma-Aldrich, St. Louis, MO, USA) (i.e., 25, 24, 20, 15, 5 g) to make a total mass of 25 g (each mixture). Samples were labeled based on treatment temperature and composition as follows: SC (native starch only), DC (date seed powder only), M1–M5 (starch–date seed composites treated at 40 °C with 1, 5, 10, 15, and 20 g of date seed powder, respectively), and N1–N5 (starch–date seed composites treated at 70 °C with the same respective compositions). Date seed powder without starch (DC) was considered as a control. The incorporation levels of date seed powder (0, 1, 5, 10, and 20 g) were selected based on previous studies reporting a wide range of usage depending on the application. In food systems, incorporation levels typically range from 2% to 10% in baked goods, 1% to 5% in dairy and meat products, and up to 100% in beverages such as coffee substitutes, where date seed is used as the primary ingredient. In biodegradable food packaging, incorporation levels from 5% to 20% have been used to enhance mechanical strength, antioxidant release, and barrier properties without compromising processability [9,31,42]. The starch amounts (24, 24, 20, 15, and 5 g) were adjusted accordingly to maintain a constant total mass of 25 g, allowing us to evaluate how varying the proportion of date seed affects the physicochemical and structural properties of the resulting composites. Each mixer was placed in a beaker, and then 50 g of water was added to each mixture. The beaker was then placed on a hot plate with an agitator, and the temperature was maintained at either 40 or 70 °C. Heating and mixing were conducted for 1 h under atmospheric conditions (i.e., no inert gas or vacuum) in an open laboratory environment. No controlled atmosphere chamber was used during the heating. After heating, the mixtures were allowed to cool at an ambient temperature (~25 °C) for approximately one hour. The mixtures were transferred into different glass dishes and freeze-dried (Labtron, Surrey, UK) for 72 h.

### 2.3. Chemical Analysis

Proximate composition (i.e., moisture content, fat, crude proteins, ash, and carbohydrate) was measured according to the AOAC (2020) method as described in [43]. The moisture content was measured using a vacuum oven at 70 °C for 72 h. The fat content was extracted via the Soxhlet method using diethyl ether. Protein was determined by using the Kjeldahl method. The ash content was determined by burning samples in a muffle furnace at 550 °C for 18 h (AOAC, 2020). The total carbohydrates were determined by subtracting added amounts of water, protein, fat, and ash from the total composition. All measurements were performed in triplicate.

### 2.4. Hygroscopicity

The hygroscopicity of the date seed–starch mixtures was determined by equilibrating the mixtures in a controlled humidity chamber (Binder, NY, USA) [44]. Glass beakers (20 mL) containing 0.2 g of the sample were placed in the chamber (at 30 °C and 90% relative humidity) until equilibration. Adsorption kinetics of a 0.2 g sample were used to calculate the equilibrium time. The moisture content of the equilibrated sample was used to calculate the hygroscopicity (i.e., higher moisture content means higher hygroscopicity) [45,46].

### 2.5. Water Absorption and Solubility

A centrifugation method was used to measure the water absorption or retention capacity according to the AACC method 56-11-02 (AACC, 1984). In this method, 0.5 g of the sample was mixed with 5 mL of distilled water in a Falcon tube using a vortex. The tube was kept at 20 °C for 30 min before being centrifuged at 2000 rpm for 10 min (Measuring and Scientific Equipment, California, USA). The supernatant was separated by draining the liquid above the solids. The water retained (i.e., the difference between the initial and final mass of the residue) was divided by the sample’s initial mass to calculate the water absorption [47]. The solids content of the supernatant was determined by drying the sample at 105 °C for 18 h, and the solubility was determined as g solids/L [44]. The procedure was performed in triplicate for all samples.

### 2.6. Different Scanning Calorimetry (DSC)

Differential Scanning Calorimetry was used to analyze the thermal properties, including glass transitions and the solid melting of the samples (DSC Q10, TA instrument, New Castle, DE, USA) [48]. A mechanical refrigerated cooling system was used to cool the sample up to −90 °C. A T-zero pan of 30 µL capacity was filled with a sample, sealed with a lid, and placed in the DSC chamber. An empty sealed pan was utilized as a reference. Nitrogen gas was used as a carrier gas at a flow rate of 50 mL/min. The sample (3–10 mg) was placed in the sealed aluminum pan and was cooled to −90 °C at 5 °C/min and then scanned from −90 to 250 °C at a heating rate of 10 °C/min. The heating protocol was standardized in an acceptable heating rate that assured the tracing of the thermograms (i.e., faster heating rates could be less sensitive) [49]. A shift in the thermogram line indicated a glass transition, and an endothermic peak suggested solids melting. Each sample was run in triplicate.

### 2.7. Fourier-Transform Infrared Spectroscopy (FTIR)

The sample (0.02 g) of date seed powder and developed composites were mixed with 2 g of potassium bromide (KBr) in a ceramic mortar and pestle. The homogenous mixture was used to prepare tablets using a hydraulic press (Specac, Orpington, UK) with a 10-ton pressure (i.e., 405 kPa) and 7 min pressure time. Tablets were stored in an air-tight container. FTIR spectra were obtained from 32 scans at a resolution of 4 cm^−1^ and collected in the absorbance mode from 4000 to 40 cm^−1^ using Bruker FTIR (Bruker, Ettlingen, Germany) at 20 °C. The reference used was room air [50]. For each sample, one composite was measured three times, and five composites were considered (i.e., a total of 15 replicates). The absorption at a selected wave number was determined and expressed as means ± standard deviation (SD).

### 2.8. X-Ray Diffraction (XRD)

X-ray diffraction (Rigaku, Tokyo, Japan), using Cu Kα radiation with a wavelength of λ = 1.54 Å operating at 45 kV and 40 mA, was used to determine the crystal structure of date seed powder and developed composites. Each sample was pressed uniformly in a rectangular groove of the XRD glass slide, inserted into the XRD machine, and scanned at 2θ from 10 to 90° with a scanning speed of 10°/min and a step size of 0.02°/s. Ruland crystallinity was calculated according to Ruland (1961) by dividing the areas of all crystalline peaks by the total area of the diffractogram (i.e., sum of crystalline peaks and area below the peaks, i.e., amorphous strand) [44].

### 2.9. Field Emission Scanning Electron Microscope (FESEM)

The surface morphology of date seed powder and produced composites was examined using a Field Emission Scanning Electron Microscope (JEOL, Tokyo, Japan) at 3 kV and a 10 mm working distance. To avoid the charging effect during the measurement, samples were coated with platinum (approximate thickness of 10–20 nm under vacuum pressure of 0.2 Torr) using a sputter coater (SPI, West Chester, PA, USA) [44].

### 2.10. Statistical Analysis

All analysis was carried out in triplicate. The data are expressed as mean ± standard deviation. An analysis of variance (ANOVA) and least significant difference (LSD) were used to test the significant differences between the means of the samples (*p* < 0.05) using SPSS program (version 18.0).

## 3. Results and Discussion

### 3.1. Proximate Composition

The proximate composition of date seed powder is presented in Table 1. The moisture content of date seed powder was 1.54 g/100 sample. Date seeds contain significant levels of structural polysaccharides, such as cellulose, hemicellulose, and lignin, which enhance their value as a functional and structural component in food systems. Date seeds are mainly composed of lignocellulosic biomaterials, rich in carbohydrates (i.e., insoluble fibers). The protein, fat, ash, and carbohydrate contents in this study were 4.48, 8.91, 0.89, and 84.17 g/100 g sample, respectively. These findings are consistent with prior literature reporting a protein content up to 12.5 g/100 g, fat between 3.9 and 13.8 g/100 g, and soluble fiber from 4.4 to 6.7 g/100 g, which minimizes interference in starch matrices and supports their use as reinforcing fillers [51]. Rahman et al. [52] studied the proximate composition of roasted and unroasted date seeds. The amounts of protein, fat, ash, and carbohydrates in their study were 7.08, 8.08, 0.98, and 62.31 g/100 g sample, respectively. In general, the amounts of protein, fat, ash, and carbohydrates are within the range of values of date seed powder presented in different studies [1,53,54].

### 3.2. Evaluation of Hygroscopicity Properties

The hygroscopicity, solubility, and water absorption of date seed powder and date seed–starch composites at two temperatures (40 and 70 °C) are presented in Table 2 and Table 3. The hygroscopicity values were significantly affected by the incorporation of date seed powder with starch (*p* < 0.05). The date seed powder showed a lower hygroscopicity (9.94 g/100 g sample) compared to the starch (13.39 g/100 g sample). This could be due to the composition of the starch polymer, which contains two types of molecules: amylose, formed by linear glucose chains, and amylopectin, formed by ramified glucose chains [55]. Similar findings supporting the high hygroscopicity of starch found that it can absorb moisture up to 10–17 g/100 g when equilibrated at normal atmospheric conditions [56]. Gonçalves et al. [57] studied the hygroscopicity of starch extracted from *Pinhao* seed, and the value was 24.75 g/100 g sample. The starch hygroscopicity reported in this study was lower compared to the aforementioned work, and this might be due to the different sources of starch used, as well as the extraction and purification methods. Additionally, in the case of the date seed powder, the study by Al Khalili et al. [58] also showed a higher hygroscopicity of date seed powder (i.e., 16.82 g/100 g solids) compared to our sample (i.e., 9.94 g/100 g solids). This variation could be attributed to the variety (*Mebsily*) and maturity of the date seed (i.e., *Khalas*) and differences in the particle size.

The hygroscopicity of date-seed-starch composites showed higher values compared to date seed and starch (temperature: 40 °C and 70 °C), and it decreased with the increase in date seed fraction (i.e., 19.10 to 16.80 g/100 g) at 40 °C (i.e., 18.93 to 16.04 g/100 g, respectively) at 70 °C (Table 2 and Table 3). This could be due to the hydrophobic nature of insoluble date seed [59]. In addition, considerable amounts of lipid (i.e., 8.91 g/100 g sample) and protein (4.48 g/100 g sample) in date seed powder affected their hydrophobicity [60,61]. The overall increase in the hygroscopicity of the composites as compared to date seed and starch alone could be beneficial in preparing coating formulations due to their hydration ability [62].

### 3.3. Water Solubility and Swelling Properties

The water absorption of the date seed powder (75.8 g/L) was lower than starch (88.3 g/L) (Table 2 and Table 3). Al-Khalili et al. [58] found that the date seed powder’s absorption was 85.1 g/L, as compared to this study (75.8 g/L). The absorbance (swelling) capacity of the composites showed an increasing and then decreasing trend. The highest absorbance capacity was observed for M2 (i.e., 83.01 g/L at 40 °C) and N2 (i.e., 90.30 g/L at 70 °C). This could be due to the structural building at a low date seed addition and breaking at the higher date seed content as crosslinking bonds can form between polysaccharides of date seed and starch polymers [63]. Moreover, water absorption was higher in the case of samples treated at 70 °C as compared to 40 °C. This could be due to the gelatinization of starch at 70 °C.

Starch showed lower solubility (i.e., 1.6 g/L) compared to the date seed powder (i.e., 17.8 g/L) (Table 2 and Table 3). This could be attributed to the high amylopectin content of the starch, which could retain water within its three-dimensional branched structure [60,64,65]. The solubility of date seed–starch composites showed an increasing trend with the increase in the added date seed powder (Table 2 and Table 3). This was expected because the date seed powder (control) showed a higher solubility (17.8 g/L). However, composites prepared at 70 °C showed higher solubility values (5.7 to 13.7 g/L) compared to the composites prepared at lower temperatures (0.9 to 8.9 g/L). The higher solubility of the composite prepared at 70 °C could be attributed to the gelatinization of starch granules, forming a homogenized structure that tends to absorb more water [63]. Comparable results were observed in starch films containing partially defatted rice bran (PRB) [66], where the solubility decreased at moderate PRB levels due to better filler–matrix interaction but increased at higher levels due to the disruption of the starch network [67]. Similarly, in this study, a moderate date seed addition improved the structure and water resistance, while excessive loading and heat treatment raised the solubility due to a weakened matrix integrity.

### 3.4. Surface Morphology

**Figure 1:**
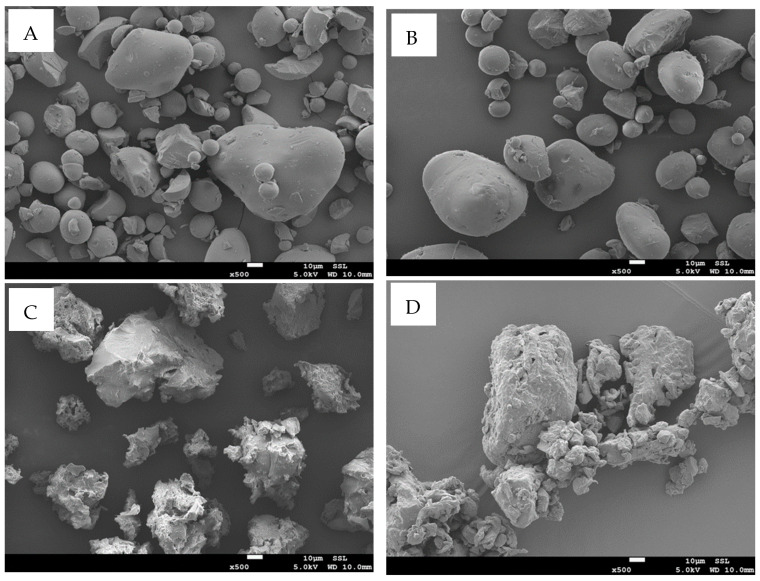
Field emission scanning electron microscope of SC (**A**), M1 (**B**), N1 (**C**), and DC (**D**). SC = starch control; DC = date seed control (untreated powder); M1 = starch–date seed composites with 1 g of date seeds and N1 = starch–date seed composites with 1 g of date seeds, respectively, at 40 and 70 °C.

The field emission scanning electron microscope (FESEM) images of the date seed powder and starch M1 and N1 (i.e., at 500 magnifications) are displayed in Figure 1. Starch (SC) showed a relatively spherical shape with a smooth surface (Figure 1A). Similarly, Sujka & Jamroz [68] observed a characteristic spherical shape with an entirely smooth surface in the native corn and rice starch. The morphology of the starch granules remained a regular spherical shape when heated at 40 °C (Figure 1B), while starch heated at 70 °C showed structural damage and a rough surface (Figure 1C). The higher-temperature damage could be due to the gelatinization process that occurs above 50 °C. Date seed powder (DC) showed agglomerated particles with a rough surface and holes on the surface (Figure 1D). Suresh et al. [69] mentioned that DC has a tightly packed structure. Similarly, Castillo et al. [70] reported that thermal processing, especially in the presence of plasticizers, leads to a progressive disruption of starch granules, causing agglomeration, surface roughness, and eventual loss of granular integrity, which supports the observed morphological changes.

**Figure 2:**
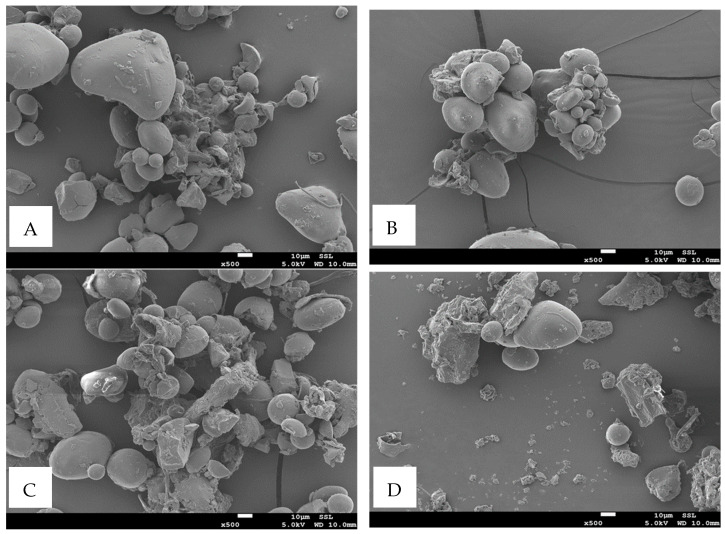
Particle morphology of starch–date seed composite prepared at 40 °C: M2 (**A**), M3 (**B**), M4 (**C**), and M5 (**D**). M2–M5 = starch–date seed composites with 5, 10, 15, and 20 g of date seeds at 40 °C.

**Figure 3:**
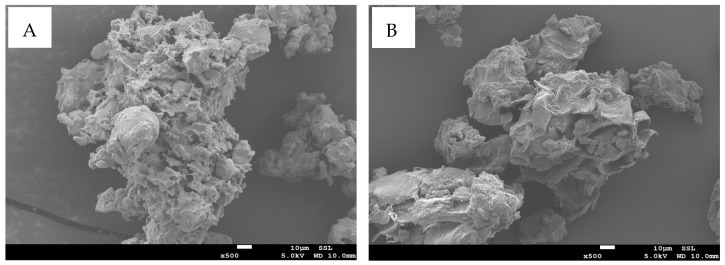

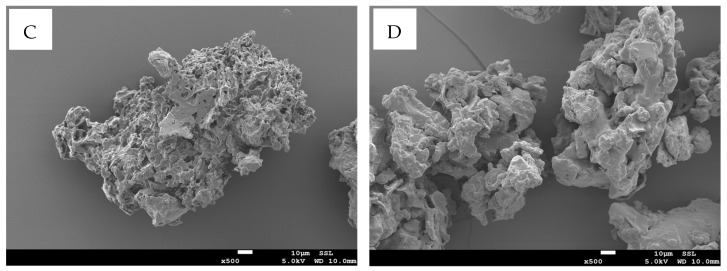
Particle morphology of starch–date seed composite prepared at 70 °C: N2 (**A**), N3 (**B**), N4 (**C**), and N5 (**C**). N2–N5 = starch–date seed composites with 5, 10, 15, and 20 g of date seeds at 70 °C.

Figure 2 shows the particle morphology of the starch–date seed composite prepared at 40 °C. It shows that the particles increased with the increase in date seed; however, the sample M4 showed the highest agglomerated particles forming a matrix (Figure 2C). In all cases, some intact starch particles could be observed. Figure 3 shows the effect of heating (i.e., 70 °C) at the different levels of added date seed. In this case, agglomerated particles with a porous structure were formed. However, in the case of M4 (Figure 3C), finer pores were observed. The agglomerated matrix was due to the gelatinization of starch at 70 °C and the formation of networks between the starch and date seed particles. Alqahtani et al. [71] incorporated date seed powder with corn starch to produce biodegradable films. They observed that date seeds produced a compact and continuous structure. This could be due to the formation of hydrogen bonds and hydrophobic interactions between the starch and date seeds [72].

### 3.5. XRD Analysis

**Figure 4:**
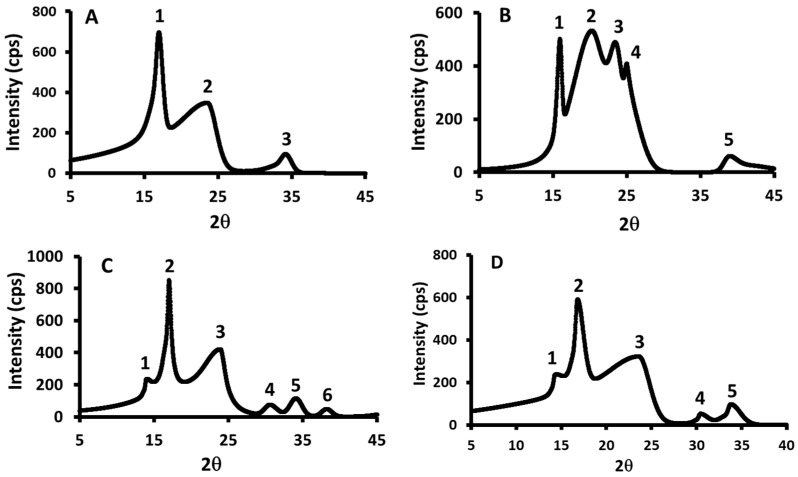
XRD micrographs for the starch (SC) (**A**), date seed (DC) (**B**), and starch–date seed composites prepared at 40 °C (sample M1) (**C**) and 70 °C (sample N1) (**D**). SC = starch control; DC = date seed control (untreated powder); M1 = starch–date seed composites with 1 g of date seeds and N1 = starch–date seed composites with 1 g of date seeds, respectively, at 40 and 70 °C.

Figure 4 shows XRD micrographs for the starch, date seed, and starch–date seed composites prepared at 40 °C (sample M1) and 70 °C (sample N1). The number of crystallites, d-value, and size of the crystallites for all composites at 40 °C and 70 °C are illustrated in Table 4 and Table 5. Starch shows three types of crystallites (2θ: 16.9, 23.5, and 34.1°), while date seeds show five types of crystallites (2θ: 15.9, 20.1, 23.5, 25.0, and 38.9°). In comparison, Pozo et al. [73] observed five types of crystallites (2θ: 15.26, 17.21, 19.75, 22.32, and 24.08°) in a starch sample. Also, Fu et al. [74] observed four strong peaks (2θ: 15, 17, 18, and 23°) for gelatinized starch. In the case of defatted date seed powder, Al-Khalili et al. [44] observed six peaks at 2θ values of 16.0, 18.4, 20.0, 24.0, 25.6, and 83.5°, respectively.

**Figure 5:**
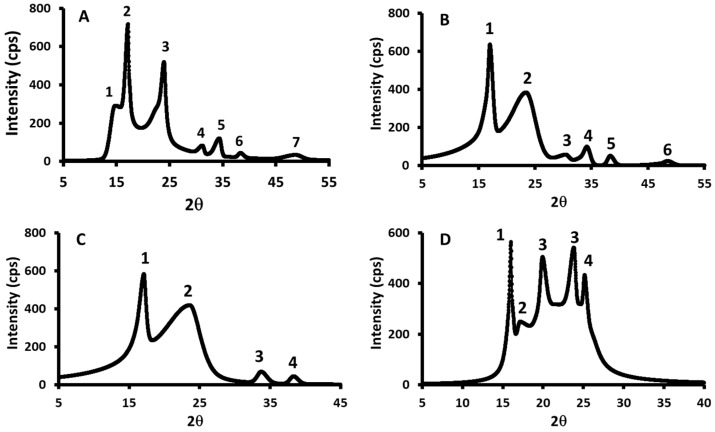
XRD micrographs for the M2 (**A**), M3 (**B**), M4 (**C**), and M5 (**D**) prepared at 40 °C. M2–M5 = starch–date seed composites with 5, 10, 15, and 20 g of date seeds at 40 °C, respectively.

**Figure 6:**
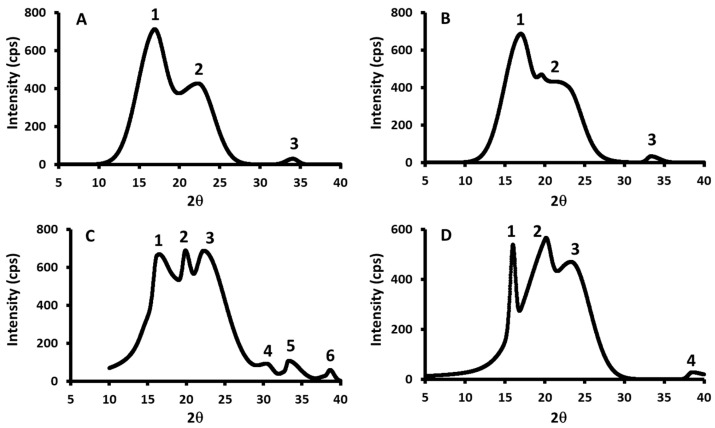
XRD micrographs for the N2 (**A**), N3 (**B**), N4 (**C**), and N5 (**D**) prepared at 70 °C. N2–N5 = starch–date seed composites with 5, 10, 15, and 20 g of date seeds at 70 °C, respectively.

Samples M1, M2, M3, and M4 showed common crystallite peaks (2*θ*: 23.5 and 38.9°) (Figure 5). However, samples containing higher amounts of date seed exhibited characteristic peaks at 2*θ*:15.9° (observed in date seed and M5) and 2*θ*:38.4° (observed in N4 and N5), which were absent in the other composites. Similarly, starch and composites with higher starch content displayed a distinct peak at 2θ:34.0°, not present in M4 and M5. Peaks at 2θ:23.5° and 33.8° were consistently observed in composites N1, N2, N3, and N4 (Figure 6). Overall, the crystallite patterns of the composites at 40 °C and 70 °C demonstrated intermediate crystallinity, falling between those of pure starch and pure date seed powder.

Table 6 presents the crystallinity of starch, date seed powder, and composites at 40 °C and 70 °C. The crystallinity of starch was higher (i.e., 40.7%) compared to the date seed powder (i.e., 17.2%). The crystalline values of the native starch granules vary from 10 to 50%, mainly depending on the botanical origin of the starch [75]. The high-crystallinity phase of the starch, compared to the seed powder, is attributed to the compact matrix that contains double helices among amylopectin chains. The amorphous region, on the other hand, is less ordered and forms interactions between amylose and amylopectin branches [76]. Nabili et al. [10] observed that a major part of the date seed sample was dominated by amorphous matter (i.e., lower crystallinity). However, Al-Khalili et al. [44] found that the crystallinity of defatted date seed was 62.6%. Also, Kruer-Zerhusen et al. [77] found that the crystallinity of cellulose samples showed different extents of digestion. Therefore, the date seed could show varied functionality.

The crystallinity of the composite M1 was 41.6%, and it reduced to 12.8% only for the M5 sample (*p* < 0.05). For composites prepared at 70 °C, a decreasing trend was observed (i.e., 24.0 to 11.3%) (Table 6). The reduction in crystallinity was due to the addition of the low-crystallinity date seed powder. However, composites prepared at 70 °C showed a greater reduction in crystallinity compared to those prepared at 40 °C. This could be due to the gelatinization process of starch, which occurs between 50 and 90 °C [76]. Starch gelatinization and general granule disruption in water are related to the starch crystallites.

The observed reduction in crystallinity is also influenced by the presence of insoluble dietary fiber from date seeds, namely cellulose, hemicellulose, and lignin, which act as rigid, non-crystalline fillers that disrupt the ordering of amylopectin helices during gelatinization and retrogradation. Such disruption limits the formation of ordered crystalline zones within the starch matrix [78]. These insoluble fibers do not integrate at the molecular level but instead create physical obstacles that prevent recrystallization of starch chains. This effect is consistent with XRD data, showing suppressed starch peaks and a reduced overall crystallinity in starch–cellulose composites.

It was interesting to see that at high date seed fractions (i.e., M5 and N5), the crystallinity did not change significantly for the treated sample at 40 °C and 70 °C (*p* < 0.05). Similar trends were reported in starch composites with coconut husk fiber [79], where a moderate fiber addition increased the crystallinity due to nucleation, but excess fiber caused aggregation, reducing the crystallinity [80,81]. In contrast, the date seed powder consistently reduced the crystallinity in this study, likely due to its lower ordering and lack of nucleating effect [82,83]. This highlights that, while both fillers alter the starch structure, their mechanisms and impacts differ.

This confirms that insoluble fiber from date seeds primarily acts as a structural disruptor and leads to the observed decrease in crystallinity. Recognizing this role is important for tailoring material properties, since a lower crystallinity often correlates with enhanced flexibility and biodegradability. Moreover, comparing these results with other fiber–starch systems in the literature provides a deeper insight into the crystallinity changes. For instance, starch composites with cellulose nanocrystals often exhibit increased crystallinity due to the nucleating effect of the crystalline cellulose, which promotes amylopectin chain alignment and recrystallization [84,85]. However, studies have shown that date seed extracts incorporated into starch-based polymeric films lead to broader, less defined XRD peaks indicative of an increased amorphous character, likely due to molecular interactions disrupting polymer chain ordering and crystalline regions [27].

This distinction underscores the importance of fiber morphology and crystallinity in dictating the composite structure. Additionally, thermal processing conditions, such as those above gelatinization temperatures, further modify these interactions by enhancing the starch’s molecular mobility, thus amplifying the disruptive effects of amorphous fibers like date seed powder, especially under mechanical or thermal treatment [86]. These comparisons emphasize that the observed decrease in crystallinity in our starch–date seed composites aligns well with findings in similar fiber–starch systems, where amorphous fiber additions lead to reduced crystallinity and altered material properties.

### 3.6. Thermal Analysis

#### 3.6.1. Starch

**Figure 7:**
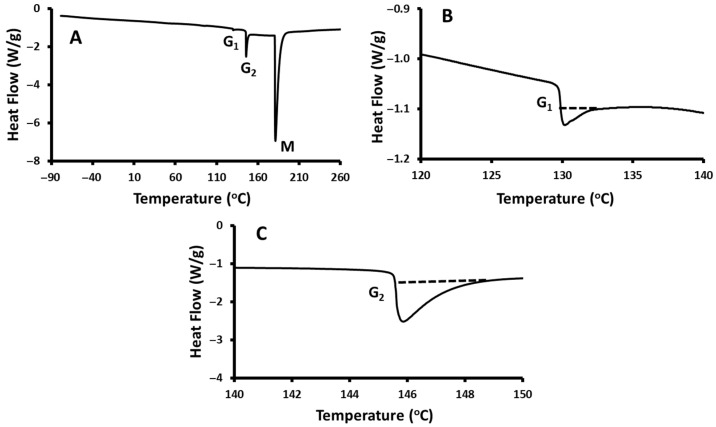
DSC thermograms of starch at a heating rate of 10 °C/min: (**A**) Full thermogram showing all thermal transitions; (**B**) first glass transition (G1); (**C**) second glass transition (G2). G1: first glass transition, G2: second glass transition, M: solid melting peak observed in the samples.

DSC thermograms of starch (moisture content: 5.3 g/100 g sample), at a heating rate of 10 °C/min, are shown in Figure 7. The thermogram showed two shifts related to glass transition (G1 and G2) and one endothermic peak presented as (M).

The onset, mid-, and end-points of the first glass transition were observed as 128.5, 128.7, and 128.9 °C, respectively, and the ∆*Cp* was 328.3 J/kg K. The second glass transition was observed at 145.5, 145.6, and 147.9 °C, respectively, with a ∆*Cp* of 1093.2 J/kg K (Table 7). The multiple glass transitions were attributed to the increase in the ∆*Cp* of cooperative volume (i.e., chain connectivity and composition fluctuations) [87]. In foods and other biomaterials, multiple glass transitions were observed for extracted starch foods [88], fish [89], and polystyrene [59,87]. Homer et al. [90] observed the first (154, 137, 161 °C) and second (149, 169, 161 °C) glass transition temperatures, respectively, for the onset, mid-point, and end-point for starch (moisture content: 6.2 g/100 g sample) at a heating rate of 10 °C/min. These findings corroborate the dual glass transitions observed in the current study, indicating that the water content and polymer compatibility likely influenced the separation of molecular relaxation domains. Similarly, Forssell et al. [91] found two distinct glass transitions in starch–glycerol–water systems, consistent with the thermal behavior exhibited by the composite formulations analyzed in this study.

The starch used in this study (*Tg* onset ~128.5 °C) shows significantly higher glass transition temperatures than those of maize (57–67 °C), waxy corn (53–59 °C), and even high-amylose G80 starch (60–73 °C) films at comparable moisture levels (~13%). The presence of insoluble dietary fibers from date seeds, primarily cellulose, hemicellulose, and lignin, influences the thermal transitions observed in the starch–date seed composites [92]. These rigid, non-thermoplastic fibers restrict the molecular mobility of starch chains, leading to modifications in glass transition temperatures (*Tg*) and the associated heat capacity (Δ*Cp*) [93]. Specifically, the increase in the first and second glass transition temperatures with higher date seed contents (Table 7 and Table 8) suggests that fiber incorporation reduces segmental polymer chain flexibility, effectively acting as physical crosslinkers or fillers that restrict starch chain relaxation [93,94]. Furthermore, the decrease in Δ*Cp* values with an increased fiber concentration indicates a reduction in the cooperative molecular motions during thermal transitions, which is consistent with a more constrained polymer matrix. Similar findings have been reported in starch–cellulose composites, where cellulose fibers reduce the starch chain mobility and alter thermal transitions due to hydrogen bonding and physical interactions, without forming covalent bonds [95]. The reduced heat capacity and elevated *Tg* values demonstrate that insoluble fibers from date seeds increase the thermal stability by limiting the starch gelatinization and retrogradation dynamics, which is important for the functional performance of these composites during thermal processing and storage [96].

The onset, mid-, and end-points of the solid melting peak of starch were 185.7, 186.0, and 197.2 °C, respectively, and the enthalpy was found as 139.50 kJ/kg. Starches from various sources have different thermal stability due to variation in the molecular dimensions, internal mobilities, and contents of amylose and long-chain amylopectin in starch [76]. Wootton and Bamunuarachchi [97] observed that the onset, mid-, and end-points of potato starch at a heating rate of 10 °C/min were 57, 72, and 87 °C, respectively. The shape of the endotherms varied between native starches and modified starches. The gelatinization onset temperatures ranged, in the case of oat starch, from 59.4 °C to 61.4 °C and from 58.4 °C to 62.2 °C for the barley starch. The differences were due to the varying amylose and amylopectin ratios [76].

#### 3.6.2. Date Seed

**Figure 8:**
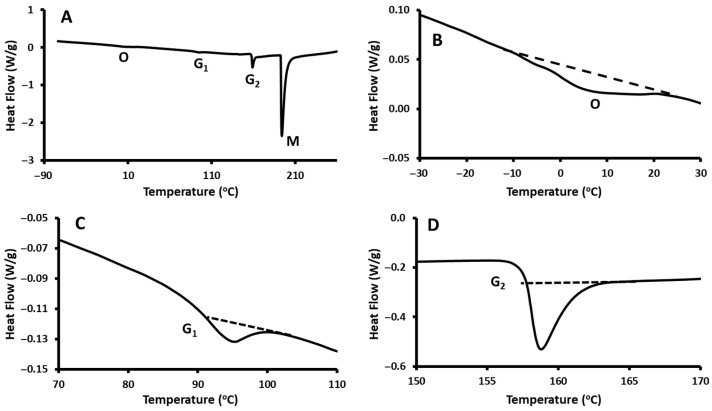
DSC thermograms of the date seed powder at a heating rate of 10 °C/min: (**A**) Full thermogram showing all thermal events; (**B**) oil melting peak; (**C**) first glass transition (G1); (**D**) second glass transition (G2). O: oil melting, G1: first glass transition, G2: second glass transition, M: solid melting peak observed in the samples.

DSC thermograms of date seed powder are illustrated in Figure 8. The thermogram showed two endothermic peaks: the first peak was due to oil melting (O), and the second peak (M) was due to the solid melting. In addition, two shifts were observed and characterized as glass transitions (G1 and G2).

The onset of the oil melting of the date seed powder was observed at −11.2 °C with a peak at 4.5 °C and ended at 16.9 °C, with the enthalpy of the peak found to be 1.9 kJ/kg sample. Similarly, Suresh et al. [69] observed the onset of the oil melting peak at −10.7 °C with a peak at 2.7 °C, ending at 17.2 °C, for the freeze-dried date seed powder (moisture content: 6.7 g/100 g sample), and the enthalpy of the peak as 2.8 kJ/kg sample. Rahman et al. [52] observed the onset of melting at −22.0 °C, peaking at −1.0 °C, ending at 16.7 °C, and the enthalpy as 4.6 kJ/kg for the roasted date seed powder. Guizani et al. [98] also observed the onset of oil melting of the date seed at −10.7 °C with a maximum slope temperature at −5.9 °C, peaking at 2.7 °C, and ending at 17.2 °C. The enthalpy of the peak was 2.8 kJ/kg sample. This peak represents the temperature at which the oil is separated from the solid matrix under the thermal treatment [99].

The onset, middle, and end temperatures of the first glass transition were observed in the case of date seed powder at 90.3, 92.0, and 94.0 °C, respectively, and the Δ*Cp* was 147.0 J/kg K. The second glass transition was observed at 156.2, 156.6, and 156.9 °C, respectively, and the Δ*Cp* was 524.0 J/kg K (Table 7). However, Rahman et al. [52] were unable to trace the glass transition shift in roasted date seed powder (moisture content: 7.0 g/100 g sample). The variation in glass transition in date seed illustrates that it is a complex biomaterial, which possesses varied rigidity due to the existence of different types of amorphous components [69]. In addition, this could be due to the heterogeneity of date seeds with a multicomponent mixture having amorphous, crystalline, and semi-crystalline phases, as well as a varied distribution of crystal lamellae thickness [100].

This thermal behavior resembles that of other fibrous agricultural residues, such as rice husk and flax fiber, which mostly exhibit dual or broad-range glass transition temperatures (approximately 70–120 °C) due to overlapping transitions of hemicellulose and lignin [101,102]. Typically, lignin shows a glass transition within the range of 100–180 °C [103]. These transitions further support the suitability of date seed powder as a thermally active reinforcement material.

The onset, peak, and end of the second endothermic peak of the date seed powder were observed at 194.5, 195.9, and 214.8 °C, respectively, and the enthalpy value was 68.5 kJ/kg (Table 9). The second endothermic peak represents the solid melting–decomposition. Al-Mawali et al. [99] reported the onset, peak, and end of solid melting–decomposition of oven-dried date seed powder (i.e., moisture content: 8.8 g/100 g sample) at 146.0, 148.3, and 155.9 °C, respectively, and the enthalpy was 125 kJ/kg. Suresh et al. [69] discovered the onset, peak, and end of solid melting–decomposition to be 59, 106, and 197 °C, respectively, with an enthalpy value of 184.0 kJ/kg. The thermal relaxations of date seed powder vary due to the difference in moisture of the date seed. Other factors, such as the variety and environmental growth conditions, could play a role.

This decomposition profile is consistent with those reported for other lignocellulosic fillers, such as coconut coir and walnut shells, which generally show degradation or melting transitions above 200 °C [104,105]. This underscores the thermal stability of date seed powder, highlighting its potential as a durable biofiller.

#### 3.6.3. Date Seed Powder–Starch Composites

Date seed powder–starch composites prepared at 40 and 70 °C showed three endothermic peaks: two glass transitions (G1 and G2) and a solid melting peak (S). However, samples with high date seed levels (i.e., M4, 10 g; M5, 20 g; and N4, 10 g; N5, 20 g) represented an additional peak (i.e., oil melting (O). Composites prepared at 40 °C showed an increased onset temperature trend in the first shift (i.e., from 132.0 to 138.3 °C), while the onset temperature in the second glass transition shift increased from 146.3 to 154.9 °C (Table 7). Similarly, for the composite prepared at 70 °C, the first glass transition temperatures increased from 133.9 to 140.4 °C and from 147.0 to 149.6 °C for the second shift (Table 8). The glass transition temperatures changed as a result of adding date seed powder at both preparation temperatures (i.e., 40 and 70 °C). For the composite prepared at 40 °C, the first Δ*Cp* showed a declining trend (i.e., from 264 to 165 J/kg K) and from 741 to 429 J/kg K at the second Δ*Cp* as the amount of added date seed powder increased. Similarly, for the composites prepared at 70 °C, the Δ*Cp* of the first shift decreased from 269 to 118 J/kg K and from 822 to 384 J/kg K for the second Δ*Cp* (Table 7 and Table 8). This was expected due to the lower Δ*Cp* value of date seed powder compared to the starch.

These trends are consistent with prior findings on starch–fiber composites, where the addition of insoluble fibers typically increases the glass transition temperatures due to restricted polymer chain mobility and decreases the Δ*Cp* due to a reduced amorphous phase mobility. For example, the incorporation of cellulose nanofiber into thermoplastic starch foam composites was found to raise the glass transition temperature (*Tg*) while improving the thermal stability, as demonstrated by Ghanbari et al. [106]. Similarly, increasing the fiber loading in cellulosic polymer composites has been shown to elevate *Tg*, indicating a rigidifying effect from the fiber reinforcement (Bahlouli et al. [107]). The increase in thermal stability with higher date seed content observed here supports the hypothesis of strong intermolecular interactions and physical restriction within the composite matrix, analogous to other starch–fiber systems.

The onset temperature of solid decomposition showed an increased trend from 182 to 193 °C for composites prepared at 40 °C and from 184 to 188 °C for composites prepared at 70 °C (Table 9). This was expected since the date seed was higher at the onset temperature compared to the starch. The enthalpy values of composites prepared at 40 and 70 °C showed a decreasing trend as the added date seed powder increased. This is probably due to the lower enthalpy of date seed powder (i.e., 68.5 kJ/kg), compared to the starch enthalpy (i.e., 139.5 kJ/kg). The lower enthalpy in the case of date seed powder indicated that structural damage requires less energy [58]. Zidan et al. [108] observed a higher thermal stability for carboxymethyl chitosan and carboxymethyl starch composite (i.e., at 450 °C) as a result of adding 2 to 8 mL of date seed extract. In addition, a higher thermal degradation temperature was observed as a result of adding 5 to 20% date seed powder to a composite membrane in a dosage-dependent manner [109]. Some studies supported that date seed powder increased composites’ thermal stability as indicated by higher glass transitions and the solid-melting temperature.

A similar increase in the thermal stability was reported when coconut fibers were added to thermoplastic cassava starch/beeswax composites. As the fiber content increased from 0 to 30%, the onset of degradation also occurred earlier, and the final residue increased from 5.9% to 14.1%, indicating enhanced thermal resistance and stronger matrix–fiber interaction [110]. Other studies showed an inconsistent effect on thermal stability when date seeds (i.e., powder or extract) were added. For instance, when a 5% date seed powder was added to a carboxymethylcellulose-based composite, the glass transition temperature increased. However, when the added amount was increased to 10%, the thermal degradation was higher (lower glass transition temperature) [62]. Moreover, Zeng et al. [111] found no change in thermal resistance of packaging papers coated with date seed oil at 2 to 5%. Overall, in the literature, the inconsistent thermal stability (degradation) trend in composites with date seeds added could be due to several reasons, such as the treatment conditions of date seeds, the extract type, the added level, and the formulation method of the composites (i.e., the homogeneity of the matrix).

### 3.7. FTIR Analysis

**Figure 9:**
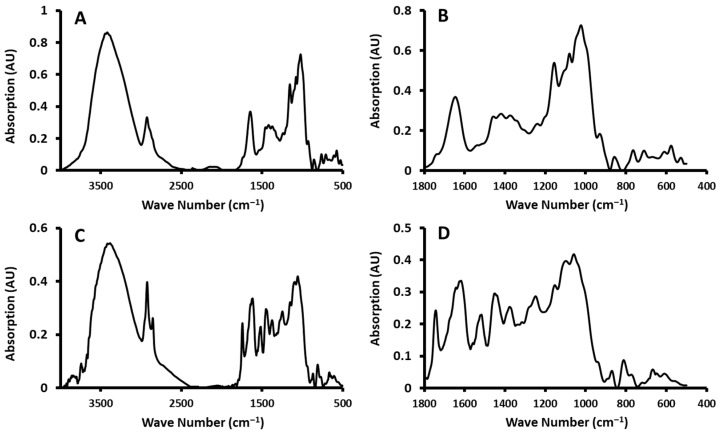
Typical FTIR spectra of native starch (**A**) and its magnified section ((**B**) 1800–400 cm^−1^) and date seed powder (**C**) and its corresponding magnified section ((**D**) 1800–400 cm^−1^).

Figure 9 shows the FTIR spectra of native starch (A) and its magnified section (B), as well as date seed powder (C) and its corresponding magnified section (D). FTIR spectra (i.e., intensity values at specific wave numbers) of date seed powder, starch, and composites prepared at temperatures of 40 and 70 °C are presented in Table 10 and Table 11.

The peak at 3421 cm^−1^ indicates the OH group stretching of date seeds. In the case of defatted date seeds, it was observed at 3422 cm^−1^ [112] and 3325 cm^−1^ [44]. Hittini et al. [113] observed the same band (i.e., 3412 cm^−1^) for a composite containing date seed powder and polystyrene. Pozo et al. [73] observed an OH stretching band of starch ranging between 3800 and 3000 cm^−1^, and it depended on the source of the starch.

The band intensity of starch at this wave number (i.e., 3421 cm^−1^) was higher than the date seed powder’s (Figure 9). This could be related to the higher density of strong hydrogen-bonding interactions in starches [114]. Also, this could be due to starch containing more OH groups between double helices [73]. According to the composites prepared at 40 °C, there were negligible changes in the intensity values with the addition of date seeds (Table 10). At the 70 °C treatment, the samples N3 and N5 showed the highest intensity compared to other composites (Table 11). This indicated that OH stretching did not vary monotonously with the increase in date seeds in the composites, suggesting temperature-enhanced interactions between date seeds and starch.

Date seed powder showed another two bands at 2930 and 2853 cm^−1^ and one band in the case of starch (i.e., 2853 cm^−1^). These peaks were related to asymmetric and symmetric stretching vibrations of CH, respectively. Similar findings were observed by Khwaldia et al. [115]. The peaks were observed at 2925 and 2853 cm^−1^ in the case of date seed powder; however, the intensities were present but suppressed for the second peak (2926, 1597 cm^−1^) when date seed powder was incorporated into the alginate composites. Likewise, two bands were observed (2926 and 2848 cm^−1^) for a composite containing date seed powder and polystyrene [113]. Lawal et al. [62] attributed the absorption band around 2926 cm^−1^ to the C-H bonds in the case of date seed powder–carboxymethyl cellulose composites. The same stretching band of C-H was observed at 2920–2870 cm^−1^ for the membrane composed of date seed powder, oil, and solvent [109]. The intensities of these bands of the composites prepared at 40 and 70 °C were increased as the amount of added date seed powder increased. In the case of composites with higher amounts of date seeds (i.e., M4, 10 g; M5, 20 g; and N4, 10 g; N5, 20 g) prepared at temperatures of 40 and 70 °C, two bands were observed, but their intensities were lower compared to the date seed powder (Table 10 and Table 11). This observation is novel, as it highlights the attenuated CH-stretching intensities in composite spectra compared to the pure date seed spectrum, likely due to matrix embedding or partial interaction.

The band at 1744 cm^−1^ corresponded to the stretching vibration of the C=O. Date seeds showed higher intensity compared to starch. The intensity values increased with the addition of date seeds to the composites at 40 and 70 °C (Table 10 and Table 11). These bands showed higher intensities, particularly for the composites prepared at 70 °C compared to those prepared at 40 °C. This could be related to the higher interaction of date seeds and starch at high temperatures. This provides new insight into temperature-dependent interactions involving C=O stretching in fatty acid ester groups, which are intensified in starch–date seed composites, especially at 70 °C. This band was related to the stretching vibration of the C=O in the fatty acid ester group, when date seed oil (2–8%) was added to coat the packaging paper [111]. Similarly, Khwaldia et al. [115] attributed this band to C = O groups and implied an interaction between the polymer matrix and the date seeds through hydrogen bonds. Al-Hasni et al. [112] observed this band at 1744 cm^−1^ in the case of defatted date seed powder, and they correlated it to the C=O stretching vibration and an indication of acetyl groups. On the other hand, the appearance of this peak (i.e., 1744 cm^−1^) was attributed to the hemicellulose and lignin composition in the date seeds and was present in the composite mixture of date seed extract and alginate [115].

Date seeds showed bands at 1621, 1515, and 1460 cm^−1^, while these were missing in the case of starch. This was attributed to the asymmetric and symmetric COO stretching and C-O-C stretching. Similarly, the frequencies of peaks at 1600, 1410, and 1029 cm^−1^ were observed as a result of adding the date seed extract to the alginate composites and were attributed to the asymmetric and symmetric COO stretching and C-O-C stretching [115]. Lawal et al. [62] observed a band around 1600 cm^−1^ in the date seed powder–carboxymethyl cellulose composite, and the band was correlated to the symmetric and asymmetric stretching frequencies of the carboxylate groups. Aloui et al. [116] extracted furfural from date seeds and used it as a filler in a caseinate composite. The furfural interaction with aldehyde groups resulted in a reduction in free amine groups, which leads to a decrease in amide I and amide II groups as characterized by the bands 1638 and 1538 cm^−1^. At 1515 cm^−1^, the intensity of composites at 40 and 70 °C was increased as the amount of added date seed powder increased (Table 10 and Table 11). This trend reveals a structural integration of carboxyl functionalities from date seeds into the starch matrix, not previously detailed for such blends.

The bands of CH at 1440 and 1436 cm^−1^ for the variable-angle vibration can be characteristics of saccharides [117]. The band at 1423 cm^−1^ was observed in starch but was missing in date seed powder. Also, it was observed in the composites with a high amount of starch at 40 and 70 °C (Table 10 and Table 11). This could be related to the C-H bending of CH2 [118]. There were negligible changes in intensity values with the addition of date seeds in composites prepared at 40 °C (Table 10). However, at the 70 °C treatment, the samples N3 and N4 showed the highest intensity compared to other composites (Table 11).

The peak at 1371 cm^−1^ was related to OH bending. The peak was observed in date seed powder and composites with high levels of date seed powder prepared at 40 °C (i.e., M4, 10 g and M5, 20 g), and all composites showed this band at 70 °C (Table 10 and Table 11). Lawal et al. [62] observed two absorption bands between 1420 and 1350 cm^−1^ and correlated them to the frequency of the OH bending of the date seed powder and the composite incorporating date seed powder with carboxymethyl cellulose.

Date seed powder, starch, and their composites prepared at 40 and 70 °C showed bands at 1244 and 1159 cm^−1^. Lawal et al. [62] attributed the bands between 1155 and 1101 cm^−1^ to the C-O stretching frequencies of polysaccharides when the date seed extract was added to the carboxymethyl cellulose. The band at 1159 cm^−1^ was attributed to the characteristic C-O stretching vibrations and O-H deformation vibrations of polysaccharides [119]. At peak 1244 cm^−1^, a slight change in the intensity was observed at 40 °C (Table 10). However, an increasing trend was observed in the case of composites prepared at 70 °C (Table 11). Considering the peak at 1159 cm^−1^, at 40 °C, the intensity decreased with the increase in date seeds, while at 70 °C, it increased and then decreased with a higher amount of date seeds. This could be due to the structure building at higher temperatures and higher amounts of date seeds, causing the more rigid behavior of the functional group. This non-linear pattern suggests novel temperature–composition-dependent changes in the polysaccharide structure and flexibility. Hittini et al. [113] observed a change in the bands at 1247 and 814 cm^−1^ as a result of adding date seed powder to the polystyrene composites. However, as a result of the alkaline treatment of the date seeds, these bands were changed, which confirmed a change in the lignocellulosic composition. A peak at 933 cm^−1^ was related to the C-O stretching vibration. The intensity was decreased as the amount of date seed powder increased in composites at 40 °C (Table 10). However, at the 70 °C temperature, the intensity increased and then decreased (Table 11). The bands between 1200 and 950 cm^−1^ were correlated to the C-O stretching vibration in the case of the date seed extract [115]. Lawal et al. [62] concluded that the addition of date seeds showed some changes in the locations or intensities of the FTIR spectrum peak; however, these changes could be insignificant in some cases.

In summary, the FTIR results indicate that key functional group interactions, especially those related to OH, CH, C=O, and COO, exhibited distinct changes in intensity and, in some cases, band emergence or suppression, depending on the temperature and the proportion of date seed powder. These results enhance the understanding of structural interactions in starch–date seed composites, particularly under thermal treatment, and suggest features not explicitly reported in prior studies.

## 4. Implications of Findings for Food Packaging and Nutritional Applications

**Figure 10:**
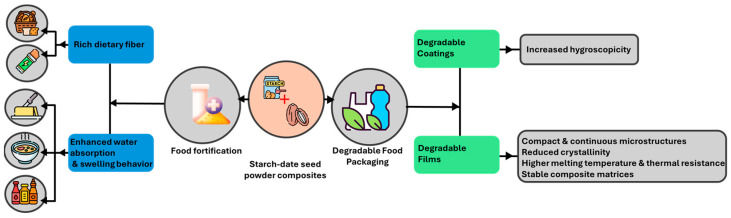
Potential applications of starch–date seed powder composites in food packaging and nutritional fortification.

The comprehensive analysis of starch–date seed powder composites revealed several key functional properties with significant implications for their potential use in biodegradable food packaging and potential nutritional fortification, as illustrated in Figure 10.

Starch–date seed powder composites may serve as fiber-enriching ingredients or binders in baked goods and energy bars, given their high dietary fiber content (Section 3.1), with total carbohydrate reaching 84.17 g/100 g (Table 1) [120,121]. Their enhanced water absorption and swelling behaviors (Section 3.3) suggest a potential use as texture modifiers or hydration regulators in formulations such as soups, sauces, dressings, and butter [122,123,124]. For example, at 70 °C, starch–date seed composites N1–N5 absorbed water between 85.2 and 90.3 g/L, with solubility increasing from 5.7 to 13.7 g/L as the date seed content increased (Table 3). This behavior is advantageous in hydration-sensitive food matrices.

The improved solubility of the composites facilitates better water interaction and dispersion within food formulations. Consequently, they are well-suited for instant or powdered products, such as soups and protein drinks, where rapid and complete dissolution is essential to ensure proper functionality and consumer acceptance [125]. In addition, they show strong potential for use in hydrated or emulsified systems, including sauces and dressings, where maintaining consistent texture and physical stability is crucial [123,126].

In addition, FTIR findings indicate molecular compatibility between starch and date seed powder (Section 3.7), which could support their incorporation into stable, functional food ingredients. Collectively, these properties point to the possible application of these composites in food products requiring fiber enhancement, controlled hydration, and structural stability [127,128,129,130,131]. The incorporation of date seed powder into starch-based composites yielded a range of structural, thermal, and chemical modifications that offer potential applications in food packaging. Each characterization technique provided insights into how these materials can be optimized for specific performance qualities. For instance, the enhanced hygroscopicity observed in starch–date seed composites (Section 3.2) indicates a potential advantage for their use as edible coatings. At 40 °C, the hygroscopicity of starch–date seed composites M1–M5 ranged from 19.1 to 16.8 g/100 g, exceeding both the starch control (13.4 g/100 g) and the untreated date seed (9.9 g/100 g) (Table 2). A similar trend was observed at 70 °C, with values from 19.0 to 16.0 g/100 g (Table 3), suggesting their potential to retain moisture in coated food systems. The increased hygroscopicity enables these composites to effectively retain moisture, making them well-suited for moisture-retentive films and coatings that help preserve the texture, freshness, and shelf life of perishable foods in packaging applications [132]. This supports the development of moisture-retentive films, which are crucial for preserving the texture and shelf life of perishable foods. This agrees with Chen et al. [133], who demonstrated that starch-based mulching films with enhanced hygroscopicity and compact structure offered excellent water retention and degradability, contributing to the environmental and functional performance in applied settings. In addition, the formation of compact and continuous microstructures in starch–date seed composites (Section 3.4), particularly at 70 °C, suggests improved film integrity. This morphological enhancement is known to correlate with better barrier and mechanical properties, which are essential for biodegradable packaging, as also emphasized in recent reviews on polymeric nanocomposites [134].

The XRD analysis (Section 3.5) revealed notable reductions in the crystallinity and variations in crystallite size with the addition of the date seed powder and thermal treatment. For instance, the starch control (SC) exhibited a crystallite size of 66.52 Å at 2*θ* = 16.95°, while M5 (20 g of date seeds at 40 °C) reached 175.83 Å at 2*θ* = 15.97°, indicating increased disorder (Table 4). At 70 °C, the crystallite size also increased, with N5 showing 111.38 Å at the same 2*θ*. Conversely, the crystallite size at 2*θ* ≈ 23.6° dropped from 10.69 Å in SC to 8.85 Å in N3 at 70 °C (Table 5), highlighting enhanced amorphous characteristics. This structural interpretation is further supported by the calculated crystallinity and amorphous content (Table 6). At 40 °C, the crystallinity decreased from 41.6% in M1 to 12.8% in M5 as the date seed levels increased, while the amorphous content increased to 87.1%. At 70 °C, this effect was even more pronounced, with N1 having 24.0% crystallinity and N5 dropping to just 11.3%, corresponding to a peak amorphous level of 88.7%. These findings confirm the destabilization of crystalline domains and increased flexibility, which may enhance the biodegradation rates and film adaptability. This behavior aligns with previous findings where reduced crystallinity facilitated processing and disintegration in biodegradable films [135].

Further, the thermal analysis (Section 3.6) demonstrated the influence of the date seed powder on thermal transitions, including increased melting temperatures and reduced enthalpy with a higher filler content. The DSC results showed that the first glass transition temperature (*Tgp*) increased from 128.7 °C in starch (SC) to 138.6 °C in M5 (Table 7), while the second glass transition also shifted upward from 145.6 °C to 154.8 °C. However, the corresponding Δ*Cp* values decreased significantly from 328 to 165 J/kg·K (first *Tg*) and from 1093 to 429 J/kg·K (second *Tg*), indicating a reduced heat capacity and increased thermal resistance. This trend continued at 70 °C (Table 8), where *Tgp* reached 140.5 °C in N5, and Δ*Cp* dropped to 118 J/kg·K (first *Tg*) and 382 J/kg·K (second *Tg*), confirming the enhanced thermal stability of composites at elevated temperatures. The solid-melting characteristics (Table 9) further support these observations, showing that the melting peak temperatures (*Tmp*) of starch–date seed composites varied with the date seed content and temperature treatment. At 40 °C, the *Tmp* ranged from 182 °C (M1) to 195 °C (M3, M4), while the enthalpy of melting (*ΔHm*) decreased from 163 kJ/kg in M1 to 88 kJ/kg in M5, indicating a reduced energy requirement for phase transitions with higher filler loads. At 70 °C, the *Tmp* fluctuated between 174 °C (N4) and 188 °C (N5), with the *ΔHm* values ranging from 130 to 159 kJ/kg, reflecting the composites’ enhanced thermal stability relative to the date seed powder alone (69 kJ/kg). A similar trend was reported for PLA-based composites incorporated with plant-based biomass fillers, where a higher filler content led to an increased melting temperature and enhanced thermal stability due to the formation of thermally resistant char layers and restricted polymer chain mobility during degradation [136].

With regard to the FTIR analysis, Section 3.7 confirmed multiple molecular interactions between starch and date seed powder, especially through OH and CH stretching, C=O vibrations, and carboxylate-related peaks. These findings are consistent with prior studies, where FTIR peak shifts and intensifications, particularly in the 2900–3500 cm^−1^ and 1740 cm^−1^ regions, were associated with hydrogen bonding and the integration of date seed components into polymer matrices [27,115,116]. Such chemical interactions indicate strong matrix–filler compatibility, contributing to the structural cohesion and functional stability of biodegradable composite films [109,136].

Thus, the starch–date seed powder composites exhibit properties that may support two key applications: biodegradable food packaging, due to their compact structure, thermal stability, and barrier traits; and functional food use, given their fiber content, hydration capacity, and molecular compatibility. However, further studies are needed to evaluate their sensory, nutritional, and functional performance in real food systems.

## 5. Conclusions

This study demonstrated that date seed powder–starch composites possess functional and structural properties beneficial for food applications, consistent with previous reports on lignocellulosic fiber incorporation in starch matrices. The addition of date seed powder enhanced fiber content and altered hygroscopicity, swelling, and solubility, with significant temperature-dependent effects observed between the two thermal treatments at 40 °C and 70 °C, reflecting differences in starch gelatinization and composite behavior under moderate and elevated heat. The microscopic analysis revealed particle agglomeration and network formation, confirming starch granule disruption and matrix formation upon heating. The crystallinity decreased with higher date seed contents, indicating starch structure disruption and increased amorphous regions. The thermal analysis showed increased glass transition and decomposition temperatures with the date seed, indicating improved composite stability. The FTIR confirmed strengthened hydrogen-bonding and molecular interactions, supporting the integration of starch and phenolic compounds in the date seed powder. These structural and functional modifications highlight the composites’ potential to enhance the texture, matrix integrity, and ingredient performance in complex food systems. Testing their integration into real formulations, such as baked goods, ready-to-eat snacks, meat analogs, and dairy alternatives, would provide valuable insights into their applicability and functionality across diverse product matrices. Moreover, future studies could also consider mechanical testing (e.g., tensile strength, elongation at break, elasticity, modulus, viscosity, flow behavior, and process stability) to evaluate the structural integrity and application potential of the composites. Taken together, these findings advance the scientific understanding of date seed powder as a functional component in starch-based composites, reinforcing its potential for sustainable innovation in future food product formulation.

## Figures and Tables

**Table 1:** Proximate composition of date seed powder (*Phoenix dactylifera* L.).

Composition	g/100 g of Date Seeds
Moisture	1.54 ± 0.01
Fat	8.91 ± 0.05
Ash	0.98 ± 0.14
Protein	4.48 ± 0.05
Total Carbohydrate	84.17 ± 0.16

**Table 2:** Hygroscopicity, solubility, and water absorption of untreated date seed powder (control), starch, and starch–date seed composites treated at 40 °C.

Sample	Hygroscopicity (g/100 g Sample)	Solubility (g/L)	Absorption (g/L)
SC	13.4 ± 0.1 ^b^	1.6 ± 0.3 ^a^	88.3 ± 0.2 ^e^
DC	9.9 ± 0.3 ^a^	17.8 ± 1.3 ^e^	75.7 ± 1.5 ^a^
M1	19.1 ± 0.1 ^f^	0.9 ± 0.1 ^a^	80.4 ± 0.8 ^cd^
M2	18.9 ± 0.3 ^ef^	1.1 ± 0.1 ^a^	83.0 ± 0.3 ^d^
M3	18.4 ± 0.2 ^e^	3.1 ± 0.1 ^b^	81.3 ± 0.1 ^cd^
M4	17.8 ± 0.6 ^d^	5.4 ± 0.1 ^c^	77.8 ± 0.7 ^bc^
M5	16.8 ± 0.1 ^c^	8.9 ± 0.2 ^d^	77.0 ± 0.2 ^ab^

SC = starch control; DC = date seed powder; M1–M5 = starch–date seed composites with 1, 5, 10, 15, and 20 g of date seeds at 40 °C. Values in the same column followed by different superscript letters are significantly different at *p* < 0.05.

**Table 3:** Hygroscopicity, solubility, and water absorption of untreated date seed powder (control), starch, and starch–date seed composites treated at 70 °C.

Sample	Hygroscopicity (g/100 g Sample)	Solubility (g/L)	Absorption (g/L)
SC	13.4 ± 0.1 ^b^	1.6 ± 0.3 ^a^	88.3 ± 2.0 ^c^
DC	9.9 ± 0.3 ^a^	17.8 ± 1.3 ^f^	75.8 ± 2.0 ^a^
N1	19.0 ± 0.1 ^d^	5.7 ± 0.1 ^c^	87.9 ± 0.1 ^c^
N2	18.7 ± 0.6 ^d^	4.8 ± 0.1 ^b^	90.3 ± 0.1 ^d^
N3	18.1 ± 0.1 ^d^	8.9 ± 0.1 ^d^	86.6 ± 1.0 ^bc^
N4	16.9 ± 0.9 ^c^	10.3 ± 0.3 ^d^	86.7 ± 0.7 ^bc^
N5	16.0 ± 0.2 ^c^	13.7 ± 0.4 ^e^	85.2 ± 0.3 ^b^

SC = starch control; DC = date seed powder; N1–N5 = starch–date seed composites with 1, 5, 10, 15, and 20 g of date seeds at 70 °C. Values in the same column followed by different superscript letters are significantly different at *p* < 0.05.

**Table 4:** X-ray diffraction (XRD) analysis of starch, date seed powder, and starch–date seed composite samples treated at 40 °C.

Sample	Peak No.	2*θ* (°)	D-Spacing (Å)	Peak Height (cps)	Crystallite Size (Å)
SC	1	16.95	5.22	351.42	66.52
	2	23.56	3.77	231.05	10.69
	3	34.12	2.62	62.76	41.54
DC	1	15.93	5.55	232.66	115.71
	2	20.17	4.39	341.53	16.86
	3	23.58	3.76	274.24	36.9
	4	25.06	3.54	180.11	35.72
	5	38.91	2.31	40.81	28.96
M1	1	13.93	6.35	70.13	71.20
	2	17.01	5.20	457.34	120.05
	3	23.91	3.71	279.39	16.74
	4	30.58	2.92	44.09	43.97
	5	34.07	2.62	74.25	45.95
	6	38.19	2.35	31.30	54.87
	7	48.38	1.87	24.85	20.50
M2	1	14.66	6.03	186.41	15.83
	2	17.17	5.15	338.54	113.02
	3	23.93	3.71	285.9	64.75
	4	31.16	2.86	29.10	73.42
	5	34.35	2.60	61.77	63.52
	6	38.29	2.34	16.51	75.42
	7	48.73	1.86	16.20	25.26
M3	1	16.97	5.21	325.99	77.41
	2	23.36	3.80	246.18	13.64
	3	30.34	2.94	30.93	20.84
	4	34.14	2.62	65.73	52.26
	5	38.29	2.34	33.56	67.47
	6	48.38	1.87	15.68	44.03
M4	1	17.07	5.18	275.88	68.06
	2	23.61	3.76	279.28	11.80
	3	33.70	2.65	40.24	54.27
	4	38.29	2.34	27.14	64.51
M5	1	15.97	5.54	261.79	175.83
	2	17.71	5.00	278.05	16.92
	3	19.99	4.43	348.17	17.88
	4	23.72	3.74	247.19	76.86
	5	24.98	3.56	185.18	38.91

SC = starch control; DC = date seed control (untreated powder); M1–M5 = starch–date seed composites with 1, 5, 10, 15, and 20 g of date seeds at 40 °C, respectively. 2*θ* = diffraction angle; d-spacing = interplanar distance; cps = counts per second.

**Table 5:** X-ray diffraction (XRD) analysis of starch, date seed powder, and starch–date seed composite samples treated at 70 °C.

Sample	Peak No.	2*θ* (°)	D-Spacing (Å)	Peak Height (cps)	Crystallite Size (Å)
SC	1	16.95	5.22	351.42	66.52
	2	23.56	3.77	231.05	10.69
	3	34.12	2.62	62.76	41.54
DC	1	15.93	5.55	232.66	115.71
	2	20.17	4.39	341.53	16.86
	3	23.58	3.76	274.24	36.9
	4	25.06	3.549	180.11	35.72
	5	38.91	2.312	40.81	28.96
N1	1	14.26	6.20	61.68	63.36
	2	16.76	5.28	285.71	72.85
	3	23.58	3.76	214.54	10.32
	4	30.37	2.93	31.05	69.78
	5	33.75	2.65	64.88	49.23
	6	48.44	1.87	20.09	21.44
N2	1	17.13	5.17	319.29	37.29
	2	23.63	3.76	299.67	13.87
	3	33.99	2.63	43.3	52.99
	4	38.36	2.34	30.71	50.75
N3	1	16.05	5.51	187.86	22.16
	2	23.59	3.76	192.46	8.85
	3	33.88	2.64	26.46	58.84
N4	1	16.38	5.40	166.74	37.08
	2	23.31	3.81	257.61	9.01
	3	33.83	2.64	7.64	77.21
	4	38.42	2.34	31.47	22.53
N5	1	15.97	5.54	228.95	111.38
	2	20.03	4.42	161.91	19.12
	3	23.29	3.81	313.96	11.7
	4	38.42	2.34	18.53	28.35
	5	15.97	5.54	228.95	111.38

SC = starch control; DC = date seed control (untreated powder); N1–N5 = starch–date seed composites with 1, 5, 10, 15, and 20 g of date seeds at 70 °C, respectively. 2*θ* = diffraction angle; d-spacing = interplanar distance; cps = counts per second.

**Table 6:** Crystallinity and amorphous characteristics of starch, date seed powder, and starch–date seed composites treated at 40 °C and 70 °C.

40 °C Treatment			70 °C Treatment		
Sample	Crystallinity (%)	Amorphous (%)	Sample	Crystallinity (%)	Amorphous (%)
SC	40.7 ± 0.8 ^b^	59.3 ± 0.8 ^a^	SC	40.7 ± 0.8 ^d^	59.3 ± 0.8 ^a^
DC	17.2 ± 3.2 ^a^	82.8 ± 3.2 ^b^	DC	17.2 ± 3.2 ^b^	82.8 ± 3.2 ^c^
M1	41.6 ± 2.2 ^b^	58.4 ± 2.2 ^a^	N1	24.0 ± 1.8 ^c^	75.9 ± 1.8 ^b^
M2	38.0 ± 4.5 ^b^	62.0 ± 4.5 ^a^	N2	19.5 ± 0.3 ^b^	80.5 ± 0.3 ^c^
M3	42.2 ± 0.1 ^b^	57.8 ± 0.1 ^a^	N3	12.9 ± 0.2 ^a^	87.1 ± 0.2 ^d^
M4	35.1 ± 2.5 ^b^	64.9 ± 2.5 ^a^	N4	11.5 ± 1.3 ^a^	88.5 ± 1.3 ^d^
M5	12.8 ± 2.4 ^a^	87.1 ± 2.5 ^b^	N5	11.3 ± 0.8 ^a^	88.7 ± 0.8 ^d^

SC = starch control; DC = date seed powder treated at both temperatures; M1–M5 = starch–date seed composites with 1, 5, 10, 15, and 20 g of date seeds at 40 °C; N1–N5 = starch–date seed composites with 1, 5, 10, 15, and 20 g of date seeds at 70 °C. Values in the same column followed by different superscript letters are significantly different at *p* < 0.05.

**Table 7:** DSC results of date seed powder, starch, and starch–date seed composite samples heated at 10 °C/min and treated at 40 °C.

Sample	First Glass Transition				Second Glass Transition			
	*T_gi_* (°C)	*T_gp_* (°C)	*T_ge_* (°C)	Δ*C_p_* (J/kg K)	*T_gi_* (°C)	*T_gp_* (°C)	*T_ge_* (°C)	Δ*C_p_* (J/kg K)
SC	128.5 ± 1.7 ^b^	128.7 ± 1.6 ^b^	128.9 ± 1.5 ^b^	328 ± 6 ^e^	145.5 ± 1.0 ^ab^	145.6 ± 0.8 ^ab^	147.9 ± 2.2 ^abc^	1093 ± 2 ^e^
DC	90.3 ± 0.1 ^a^	92.0 ± 0.3 ^a^	94.0 ± 0.9 ^a^	147 ± 8 ^a^	156.2 ± 1.9 ^c^	156.6 ± 1.8 ^c^	157.1 ± 2.0 ^c^	524 ± 6 ^b^
M1	132.0 ± 3.8 ^bc^	132.1 ± 3.8 ^bc^	132.2 ± 3.9 ^bc^	264 ± 7 ^d^	146.3 ± 4.8 ^abc^	146.4 ± 4.8 ^ab^	146.4 ± 4.8 ^ab^	741 ± 1 ^d^
M2	131.9 ± 0.7 ^bc^	131.7 ± 0.2 ^b^	131.7 ± 0.0 ^bc^	256 ± 8 ^cd^	137.4 ± 1.9 ^a^	139.1 ± 0.6 ^a^	139.4 ± 0.6 ^a^	756 ± 9 ^d^
M3	134.1 ± 1.4 ^bc^	134.2 ± 1.4 ^bc^	137.4 ± 1.4 ^e^	211 ± 8 ^bc^	149.9 ± 2.3 ^bc^	151.2 ± 4.4 ^bc^	151.7 ± 4.1 ^bc^	637 ± 12 ^c^
M4	132.5 ± 1.2 ^bc^	132.3 ± 1.5 ^bc^	132.6 ± 1.5 ^bc^	188 ± 4 ^ab^	150.3 ± 3.4 ^bc^	150.4 ± 2.2 ^bc^	150.4 ± 2.3 ^bc^	581 ± 10 ^bc^
M5	138.3 ± 0.3 ^c^	138.6 ± 0.3 ^c^	138.8 ± 0.6 ^c^	165 ± 6 ^ab^	154.9 ± 3.1 ^bc^	154.8 ± 3.0 ^bc^	154.9 ± 3.0 ^bc^	429 ± 8 ^a^

SC = starch control; DC = date seed control (untreated powder); M1–M5 = starch–date seed composites with 1, 5, 10, 15, and 20 g of date seeds, respectively. T*gi* = initial glass transition temperature; T*gp* = peak glass transition temperature; T*ge* = end glass transition temperature; Δ*Cp* = change in heat capacity. Values in the same column followed by different superscript letters are significantly different at *p* < 0.05.

**Table 8:** DSC results of date seed powder, starch, and starch–date seed composite samples heated at 10 °C/min and treated at 70 °C.

Sample	First Glass Transition				Second Glass Transition			
	*T_gi_* (°C)	*T_gp_* (°C)	*T_ge_* (°C)	Δ*C_p_* (J/kg K)	*T_gi_* (°C)	*T_gp_* (°C)	*T_ge_* (°C)	Δ*C_p_* (J/kg K)
SC	128.5 ± 1.7 ^b^	128.7 ± 1.6 ^b^	128.9 ± 1.5 ^b^	328 ± 6 ^d^	145.5 ± 1.0 ^a^	145.6 ± 0.8 ^a^	147.9 ± 2.2 ^ab^	1093 ± 2 ^e^
DC	90.3 ± 0.1 ^a^	92.0 ± 0.3 ^a^	94.0 ± 0.9 ^a^	147 ± 8 ^a^	156.2 ± 1.9 ^c^	156.5 ± 1.8 ^d^	156.9 ± 1.7 ^c^	524 ± 6 ^b^
N1	133.9 ± 2.2 ^cd^	134.2 ± 2.1 ^bc^	134.2 ± 2.2 ^bcd^	269 ± 6 ^c^	147.0 ± 1.7 ^ab^	146.8 ± 2.1 ^ab^	146.9 ± 2.0 ^ab^	822 ± 9 ^d^
N2	135.6 ± 5.3 ^de^	130.9 ± 5.3 ^b^	131.2 ± 6.2 ^cd^	215 ± 9 ^b^	147.9 ± 1.8 ^ab^	148.2 ± 1.5 ^abc^	148.3 ± 1.4 ^ab^	785 ± 1 ^d^
N3	135.9 ± 0.4 ^de^	136.0 ± 0.6 ^cd^	136.2 ± 0.5 ^de^	156 ± 3 ^e^	150.7 ± 0.6 ^b^	150.8 ± 0.7 ^c^	150.9 ± 0.7 ^b^	700 ± 6 ^c^
N4	129.3 ± 1.5 ^bc^	129.5 ± 1.7 ^b^	129.7 ± 1.7 ^bc^	144 ± 3 ^a^	144.6 ± 3.5 ^a^	146.6 ± 1.0 ^ab^	147.4 ± 0.1 ^a^	506 ± 9 ^b^
N5	140.4 ± 0.1 ^e^	140.5 ± 0.1 ^d^	140.6 ± 0.1 ^e^	118 ± 3 ^a^	149.6 ± 1.9 ^ab^	149.6 ± 1.9 ^bc^	149.8 ± 1.2 ^ab^	382 ± 4 ^a^

SC = starch control; DC = date seed powder control (untreated powder); N1–N5 = starch–date seed composites with 1, 5, 10, 15, and 20 g of date seeds at 70 °C, respectively. T*gi* = initial glass transition temperature; T*gp* = peak glass transition temperature; T*ge* = end glass transition temperature; Δ*Cp* = change in heat capacity. Values in the same column followed by different superscript letters are significantly different at *p* < 0.05.

**Table 9:** Solid-melting characteristics of date seed powder, starch, and starch–date seed composites treated at 40 °C and 70 °C.

Sample	Solids Melting				Sample	Solids Melting			
	*T_mi_* (°C)	*T_mp_* (°C)	*T_me_* (°C)	Δ*H_m_* (kJ/kg)		*T_mi_* (°C)	*T_mp_* (°C)	*T_me_* (°C)	Δ*H_m_* (kJ/kg)
SC	186 ± 5 ^ab^	186 ± 5 ^ab^	197 ± 5 ^a^	140 ± 6 ^e^	SC	186 ± 5 ^ab^	186 ± 5 ^ab^	197 ± 5 ^ab^	140 ± 6 ^c^
DC	195 ± 2 ^b^	196 ± 2 ^c^	215 ± 2 ^b^	69 ± 6 ^a^	DC	195 ± 2 ^b^	196 ± 2 ^b^	215 ± 2 ^c^	69 ± 6 ^a^
M1	182 ± 3 ^a^	182 ± 3 ^a^	202 ± 3 ^a^	163 ± 7 ^f^	N1	184 ± 8 ^ab^	184 ± 8 ^ab^	194 ± 8 ^ab^	130 ± 1 ^c^
M2	182 ± 1 ^a^	183 ± 1 ^a^	199 ± 2 ^a^	121 ± 3 ^d^	N2	179 ± 5 ^a^	179 ± 5 ^a^	192 ± 4 ^ab^	152 ± 3 ^d^
M3	193 ± 2 ^b^	195 ± 3 ^bc^	208 ± 7 ^bc^	106 ± 4 ^c^	N3	183 ± 4 ^ab^	185 ± 4 ^ab^	201 ± 2 ^b^	155 ± 2 ^d^
M4	193 ± 1 ^b^	195 ± 1 ^bc^	217 ± 6 ^b^	102 ± 2 ^c^	N4	175 ± 1 ^a^	174 ± 1 ^a^	186 ± 1 ^a^	159 ± 4 ^d^
M5	194 ± 3 ^b^	194 ± 3 ^bc^	205 ± 3 ^bc^	88 ± 1 ^b^	N5	188 ± 2 ^ab^	188 ± 3 ^ab^	198 ± 3 ^ab^	100 ± 6 ^b^

SC = starch control; DC = date seed powder control (untreated powder); M1–M5 = starch–date seed composites with 1, 5, 10, 15, and 20 g of date seeds at 40 °C; N1–N5 = starch–date seed composites with 1, 5, 10, 15, and 20 g of date seeds at 70 °C. T_mi_ = initial melting temperature; T_mp_ = peak melting temperature; T_me_ = end melting temperature; ΔH_m_ = enthalpy of melting. Values in the same column followed by different superscript letters are significantly different at *p* < 0.05.

**Table 10:** FTIR analysis of starch (SC), date seed powder (DC), and starch–date seed composite powders (M1–M5) treated at 40 °C.

Peaks	Wave Number (cm^−1^)	SC	DC	M1	M2	M3	M4	M5
1	3421	0.840 ± 0.019 ^d^	0.592 ± 0.003 ^c^	0.442 ± 0.003 ^a^	0.459 ± 0.003 ^a^	0.458 ± 0.003 ^b^	0.459 ± 0.003 ^a^	0.448 ± 0.002 ^a^
2	2931	0.324 ± 0.002 ^e^	0.460 ± 0.000 ^f^	0.255 ± 0.002 ^b^	0.226 ± 0.003 ^a^	0.276 ± 0.004 ^c^	0.281 ± 0.001 ^c^	0.307 ± 0.004 ^d^
3	2853	-	0.300 ± 0.000 ^d^	-	-	-	0.178 ± 0.009 ^b^	0.201 ± 0.003 ^c^
4	1798	-	0.036 ± 0.001 ^b^	-	-	-	0.038 ± 0.005 ^b^	0.040 ± 0.002 ^b^
5	1740	0.084 ± 0.003 ^ab^	0.278 ± 0.002 ^e^	0.073 ± 0.002 ^a^	0.109 ± 0.004 ^b^	0.152 ± 0.005 ^c^	0.150 ± 0.030 ^c^	0.212 ± 0.020 ^d^
6	1650	0.260 ± 0.003 ^b^	-	0.260 ± 0.002 ^b^	0.270 ± 0.002 ^bc^	0.314 ± 0.005 ^d^	0.265 ± 0.012 ^b^	0.291 ± 0.020 ^cd^
7	1621	-	0.397 ± 0.002 ^b^	-	-	-	-	-
8	1515	-	0.267 ± 0.002 ^d^	-	0.197 ± 0.006 ^b^	0.238 ± 0.01 ^c^	0.190 ± 0.020 ^b^	0.226 ± 0.012 ^c^
9	1460	-	0.343 ± 0.002 ^c^	-	-	-	0.258 ± 0.010 ^b^	0.258 ± 0.01 ^b^
10	1423	0.286 ± 0.003 ^d^	-	0.272 ± 0.00 ^c^	0.263 ± 0.002 ^b^	0.273 ± 0.01 ^c^	-	-
11	1371	-	0.298 ± 0.002 ^c^	-	-	-	0.226 ± 0.003 ^b^	0.223 ± 0.012 ^b^
12	1244	0.235 ± 0.000 ^b^	0.333 ± 0.002 ^c^	0.222 ± 0.001 ^ab^	0.234 ± 0.001 ^b^	0.215 ± 0.01 ^a^	0.209 ± 0.010 ^a^	0.226 ± 0.012 ^ab^
13	1159	0.540 ± 0.002 ^e^	0.364 ± 0.002 ^b^	0.447 ± 0.002 ^d^	0.429 ± 0.001 ^cd^	0.420 ± 0.01 ^c^	0.346 ± 0.021 ^b^	0.295 ± 0.002 ^a^
14	1102	-	0.459 ± 0.002 ^b^	-	-	-	-	-
15	1085	0.578 ± 0.002 ^e^	-	0.467 ± 0.002 ^d^	0.444 ± 0.002 ^c^	0.440 ± 0.01 ^c^	0.382 ± 0.010 ^b^	-
16	1058	-	0.485 ± 0.002 ^c^	-	-	-	-	0.362 ± 0.001 ^b^
17	1023	0.725 ± 0.002 ^d^	-	0.543 ± 0.002 ^c^	0.557 ± 0.003 ^c^	0.536 ± 0.010 ^c^	0.423 ± 0.040 ^b^	-
18	933	0.187 ± 0.002 ^d^	0.090 ± 0.002 ^a^	0.188 ± 0.001 ^d^	0.153 ± 0.003 ^c^	0.152 ± 0.010 ^c^	0.104 ± 0.003 ^b^	0.084 ± 0.001 ^a^
19	870	-	0.058 ± 0.002 ^c^	-	-	-	-	0.038 ± 0.001 ^b^
20	859	0.069 ± 0.002 ^de^	-	0.079 ± 0.001 ^e^	0.065 ± 0.001 ^d^	0.048 ± 0.004 ^c^	0.029 ± 0.01 ^b^	-
21	811	-	0.100 ± 0.002 ^c^	-	-	-	-	0.050 ± 0.001 ^b^
22	767	0.091 ± 0.002 ^e^	0.045 ± 0.002 ^b^	0.091 ± 0.001 ^e^	0.075 ± 0.001 ^d^	0.064 ± 0.002 ^c^	0.036 ± 0.004 ^a^	0.032 ± 0.001 ^a^
23	714	0.083 ± 0.002 ^f^	-	0.075 ± 0.00 ^e^	0.059 ± 0.001 ^d^	0.048 ± 0.002 ^c^	0.024 ± 0.006 ^b^	-
24	671	-	0.061 ± 0.002 ^cd^	0.069 ± 0.002 ^e^	0.059 ± 0.001 ^cd^	0.062 ± 0.002 ^d^	0.049 ± 0.004 ^b^	0.057 ± 0.001 ^c^
25	608	-	0.046 ± 0.002 ^c^	-	-	-	-	0.035 ± 0.002 ^b^
26	578	0.094 ± 0.002 ^e^	-	0.069 ± 0.002 ^d^	0.069 ± 0.001 ^d^	0.061 ± 0.002 ^c^	0.038 ± 0.01 ^b^	-

SC = starch control; DC = date seed control (untreated powder); M1–M5 = starch–date seed composites with 1, 5, 10, 15, and 20 g of date seeds, respectively. Values in the same row followed by different superscript letters are significantly different at *p* < 0.05.

**Table 11:** FTIR analysis of starch (SC), date seed powder (DC), and starch–date seed composite powders (N1–N5) treated at 70 °C.

Peaks	Wave Number (cm^−1^)	SC	DC	N1	N2	N3	N4	N5
1	3421	0.840 ± 0.019 ^e^	0.592 ± 0.003 ^d^	0.291 ± 0.027 ^a^	0.359 ± 0.002 ^b^	0.492 ± 0.004 ^c^	0.362 ± 0.006 ^b^	0.464 ± 0.005 ^c^
2	2931	0.324 ± 0.002 ^e^	0.460 ± 0.000 ^f^	0.140 ± 0.010 ^a^	0.185 ± 0.001 ^b^	0.275 ± 0.005 ^d^	0.219 ± 0.001 ^c^	0.334 ± 0.003 ^e^
3	2853	-	0.300 ± 0.000 ^d^	-	-	-	0.134 ± 0.001 ^b^	0.218 ± 0.003 ^c^
4	1798	-	0.036 ± 0.001 ^b^	-	-	0.050 ± 0.004 ^c^	0.058 ± 0.001 ^d^	0.059 ± 0.001 ^d^
5	1740	0.084 ± 0.003 ^a^	0.278 ± 0.002 ^g^	0.096 ± 0.004 ^b^	0.120 ± 0.003 ^c^	0.156 ± 0.004 ^d^	0.179 ± 0.002 ^e^	0.265 ± 0.002 ^f^
6	1650	0.260 ± 0.003 ^d^	-	0.214 ± 0.006 ^c^	0.265 ± 0.002 ^d^	0.316 ± 0.01 ^e^	0.274 ± 0.009 ^d^	0.033 ± 0.002 ^b^
7	1621	-	0.397 ± 0.002 ^c^	-	-	-	-	0.307 ± 0.002 ^b^
8	1515	-	0.267 ± 0.002 ^d^	-	0.215 ± 0.003 ^b^	0.239 ± 0.08 ^c^	0.232 ± 0.000 ^c^	0.276 ± 0.002 ^d^
9	1460	-	0.343 ± 0.002 ^e^	0.189 ± 0.005 ^b^	0.240 ± 0.002 ^c^	0.304 ± 0.01 ^d^	0.251 ± 0.003 ^c^	0.305 ± 0.002 ^d^
10	1423	0.286 ± 0.003 ^d^	-	0.177 ± 0.007 ^b^	0.222 ± 0.009 ^c^	0.275 ± 0.009 ^d^	0.201 ± 0.019 ^c^	-
11	1371	-	0.298 ± 0.002 ^F^	0.162 ± 0.009 ^b^	0.204 ± 0.002 ^c^	0.262 ± 0.007 ^e^	0.197 ± 0.003 ^c^	0.2454 ± 0.002 ^d^
12	1244	0.235 ± 0.001 ^d^	0.333 ± 0.002 ^e^	0.119 ± 0.005 ^a^	0.155 ± 0.002 ^b^	0.215 ± 0.01 ^c^	0.156 ± 0.003 ^b^	0.230 ± 0.002 ^d^
13	1159	0.540 ± 0.002 ^f^	0.364 ± 0.002 ^d^	0.244 ± 0.016 ^a^	0.316 ± 0.002 ^b^	0.423 ± 0.01 ^e^	0.298 ± 0.004 ^b^	0.342 ± 0.002 ^c^
14	1102	-	0.459 ± 0.002 ^c^	-	-	-	-	0.347 ± 0.002 ^c^
15	1085	0.578 ± 0.002 ^g^	-	0.256 ± 0.02 ^b^	0.341 ± 0.002 ^d^	0.446 ± 0.001 ^f^	0.309 ± 0.006 ^c^	0.380 ± 0.002 ^e^
16	1058	-	0.485 ± 0.002 ^b^	-	-	-	-	-
17	1023	0.725 ± 0.002 ^f^	-	0.314 ± 0.023 ^b^	0.403 ± 0.002 ^c^	0.535 ± 0.01 ^e^	0.380 ± 0.006 ^c^	0.437 ± 0.002 ^d^
18	933	0.187 ± 0.002 ^e^	0.090 ± 0.002 ^ab^	0.095 ± 0.006 ^ab^	0.123 ± 0.001 ^c^	0.148 ± 0.006 ^d^	0.098 ± 0.001 ^b^	0.088 ± 0.002 ^a^
19	870	-	0.058 ± 0.002 ^c^	-	-	-	-	0.038 ± 0.002 ^b^
20	859	0.069 ± 0.002 ^e^	-	0.034 ± 0.003 ^c^	0.0471 ± 0.001 ^d^	0.049 ± 0.003 ^d^	0.028 ± 0.001 ^b^	-
21	811	-	0.100 ± 0.002 ^c^	-	-	- ^a^	-	0.047 ± 0.002 ^b^
22	767	0.091 ± 0.002 ^d^	0.045 ± 0.002 ^b^	0.045 ± 0.003 ^b^	0.061 ± 0.001 ^c^	0.062 ± 0.003 ^c^	0.033 ± 0.001 ^a^	0.032 ± 0.002 ^a^
23	714	0.083 ± 0.002 ^f^	-	0.039 ± 0.003 ^c^	0.054 ± 0.000 ^e^	0.048 ± 0.003 ^d^	0.029 ± 0.001 ^b^	-
24	671	-	0.061 ± 0.002 ^c^	0.053 ± 0.002 ^b^	0.071 ± 0.001 ^d^	0.062 ± 0.003 ^c^	0.054 ± 0.001 ^b^	0.064 ± 0.002 ^c^
25	608	-	0.046 ± 0.002 ^d^	0.028 ± 0.003 ^b^	0.044 ± 0.001 ^cd^	0.042 ± 0.003 ^cd^	0.031 ± 0.001 ^b^	0.0402 ± 0.002 ^c^
26	578	0.094 ± 0.002 ^d^	-	0.038 ± 0.003 ^b^	0.062 ± 0.0004 ^c^	0.059 ± 0.003 ^c^	0.039 ± 0.001 ^b^	-

SC = starch control; DC = date seed control (untreated powder); N1–N5 = starch–date seed composites with 1, 5, 10, 15, and 20 g date seed, respectively. Values in the same row followed by different superscript letters are significantly different at *p* < 0.05.

## Data Availability

All data have been included in the manuscript.
